# Supplementary material for: White matter variability, cognition, and disorders: a systematic review
Source: Brain Struct Funct. 2021 Nov 3;227(2):529–44. doi: 10.1007/s00429-021-02382-w (PMC8844174; doi:10.1007/s00429-021-02382-w)
Supplement: Supplementary file 1 — Supplementary file1 (PDF 4155 KB) [file 429_2021_2382_MOESM1_ESM.pdf]

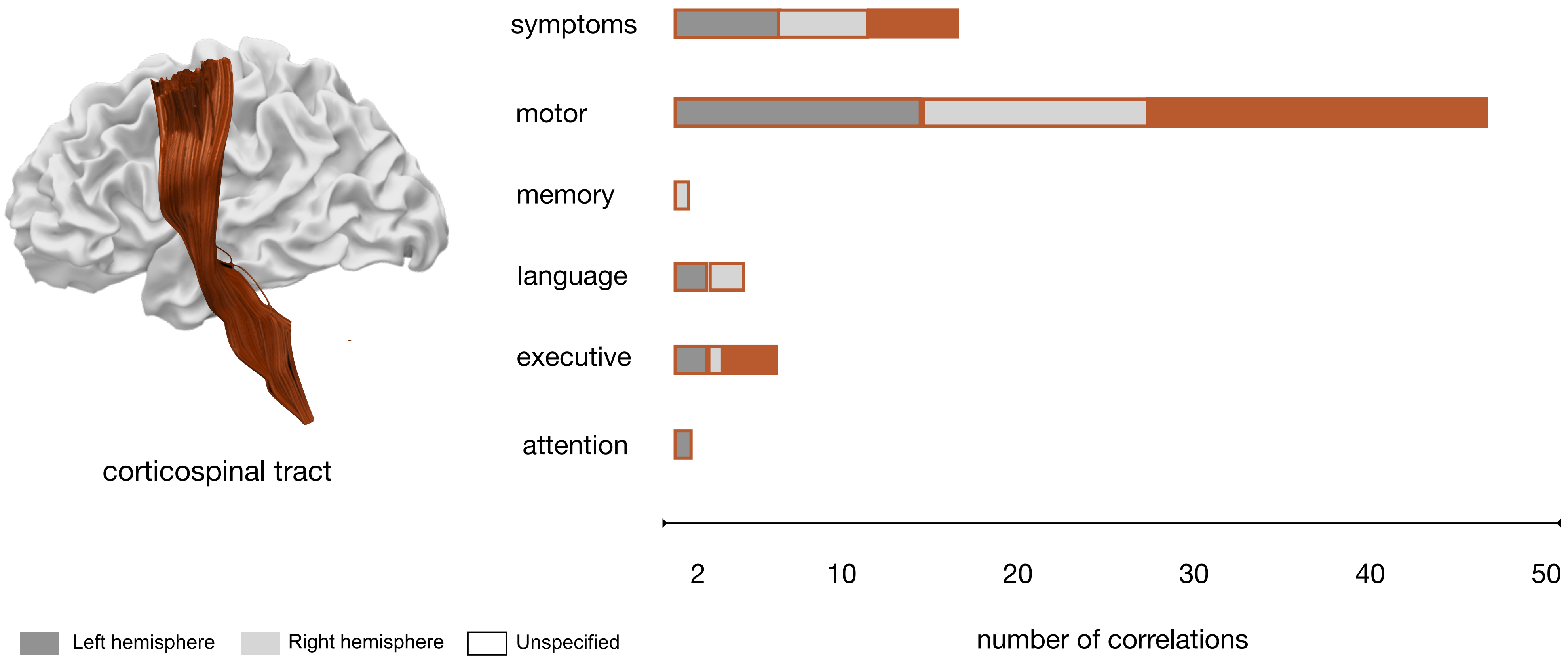
corticospinal tract
symptoms
motor
memory
language
executive
attention
2
10
20
30
40
50
number of correlations
Left hemisphere
Right hemisphere
Unspecified

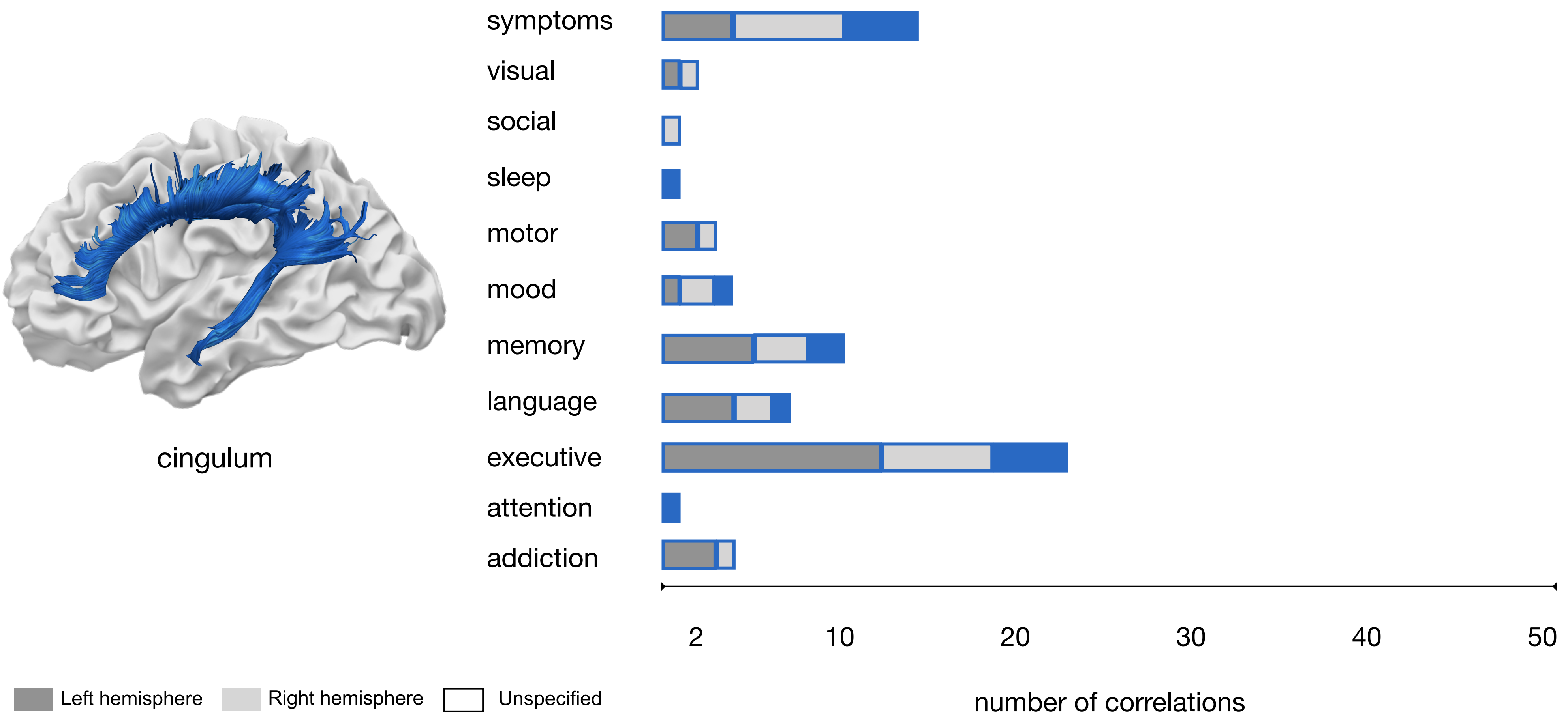
cingulum
symptoms
visual
social
sleep
motor
mood
memory
language
executive
attention
addiction
2
10
20
30
40
50
number of correlations
Left hemisphere
Right hemisphere
Unspecified

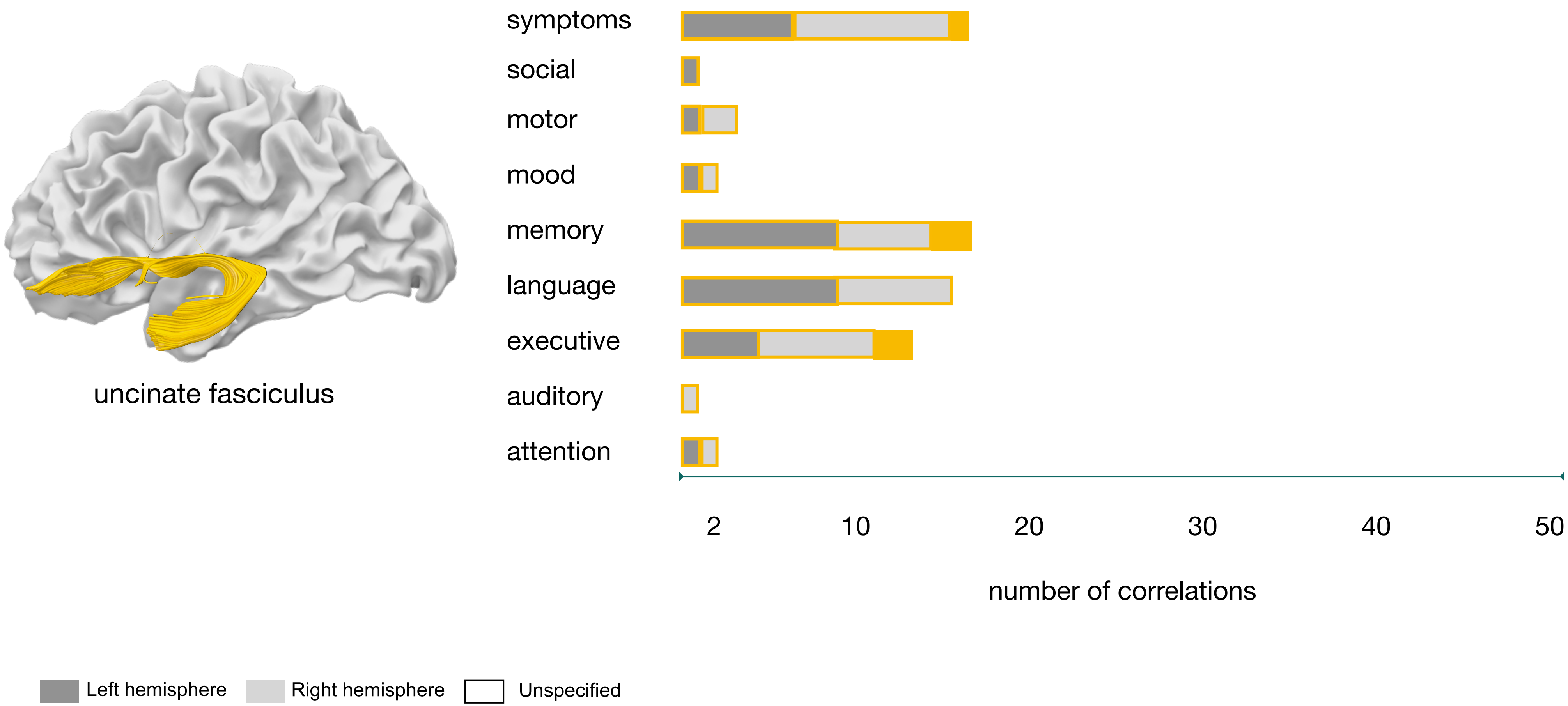
uncinate fasciculus
symptoms
social
motor
mood
memory
language
executive
auditory
attention
2
10
20
30
40
50
number of correlations
Left hemisphere
Right hemisphere
Unspecified

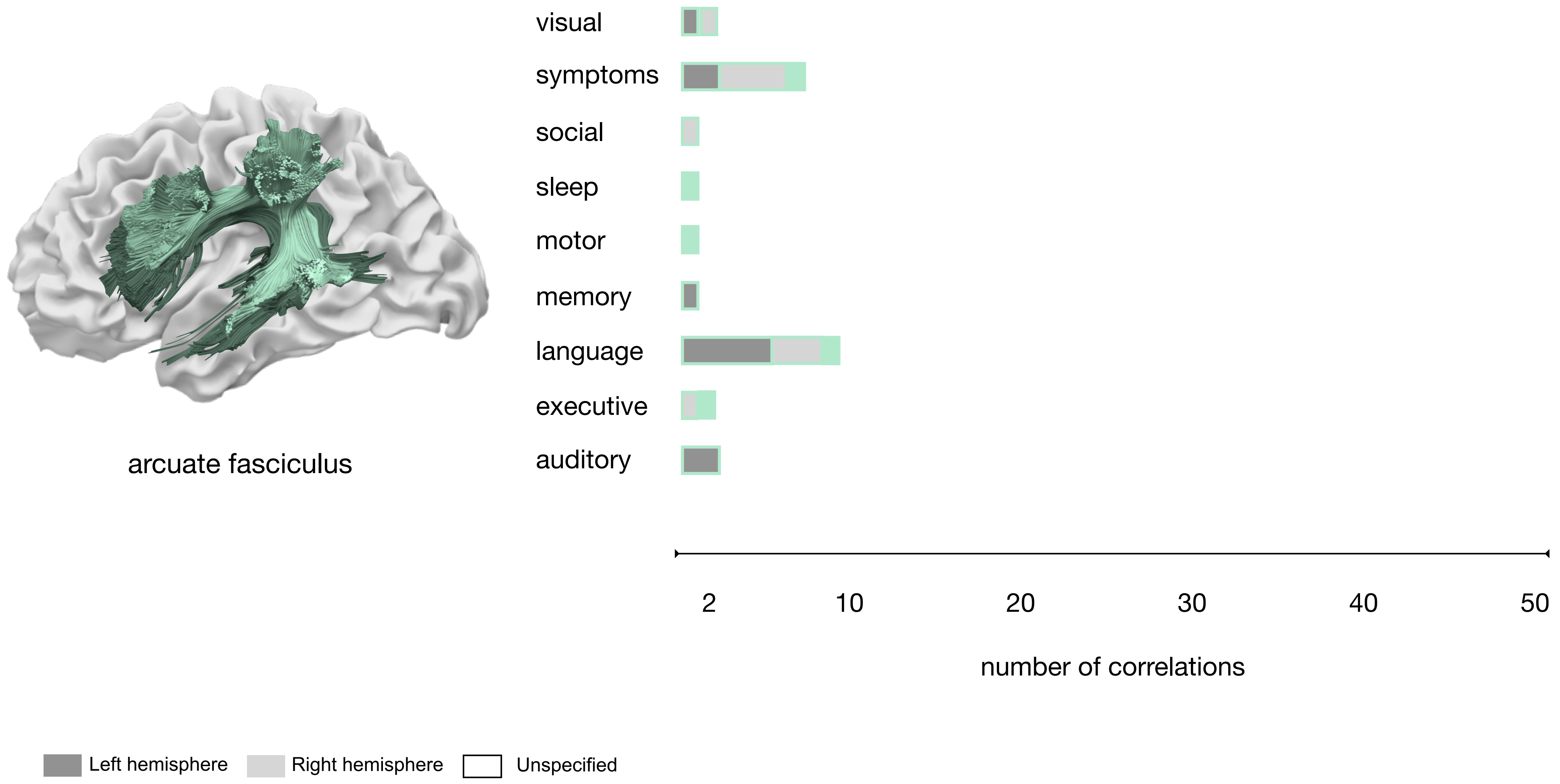
visual
symptoms
social
sleep
motor
memory
language
executive
auditory
arcuate fasciculus
2
10
20
30
40
50
number of correlations
Left hemisphere
Right hemisphere
Unspecified

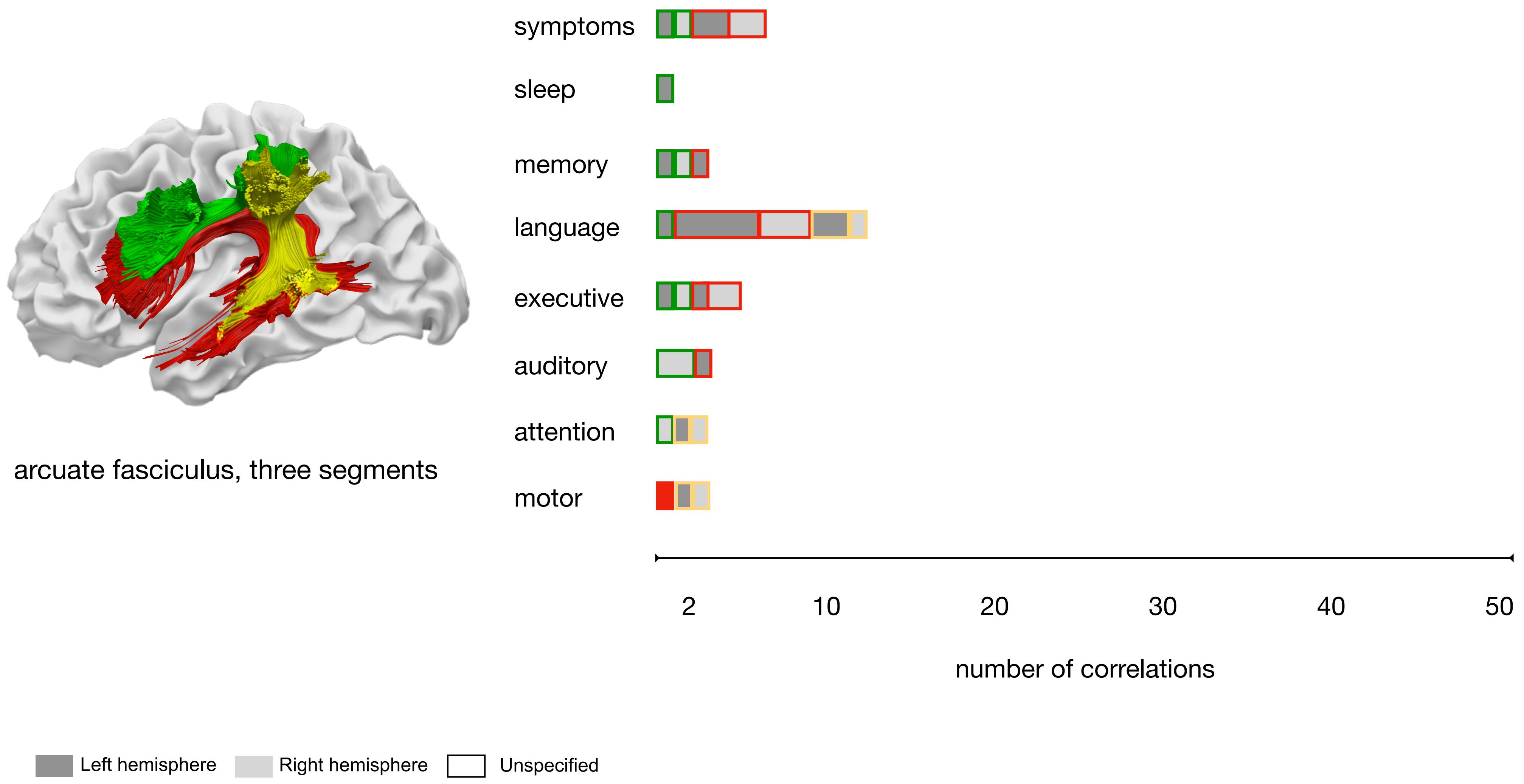

arcuate fasciculus, three segments
symptoms
sleep
memory
language
executive
auditory
attention
motor
2
10
20
30
40
50
number of correlations
Left hemisphere
Right hemisphere
Unspecified

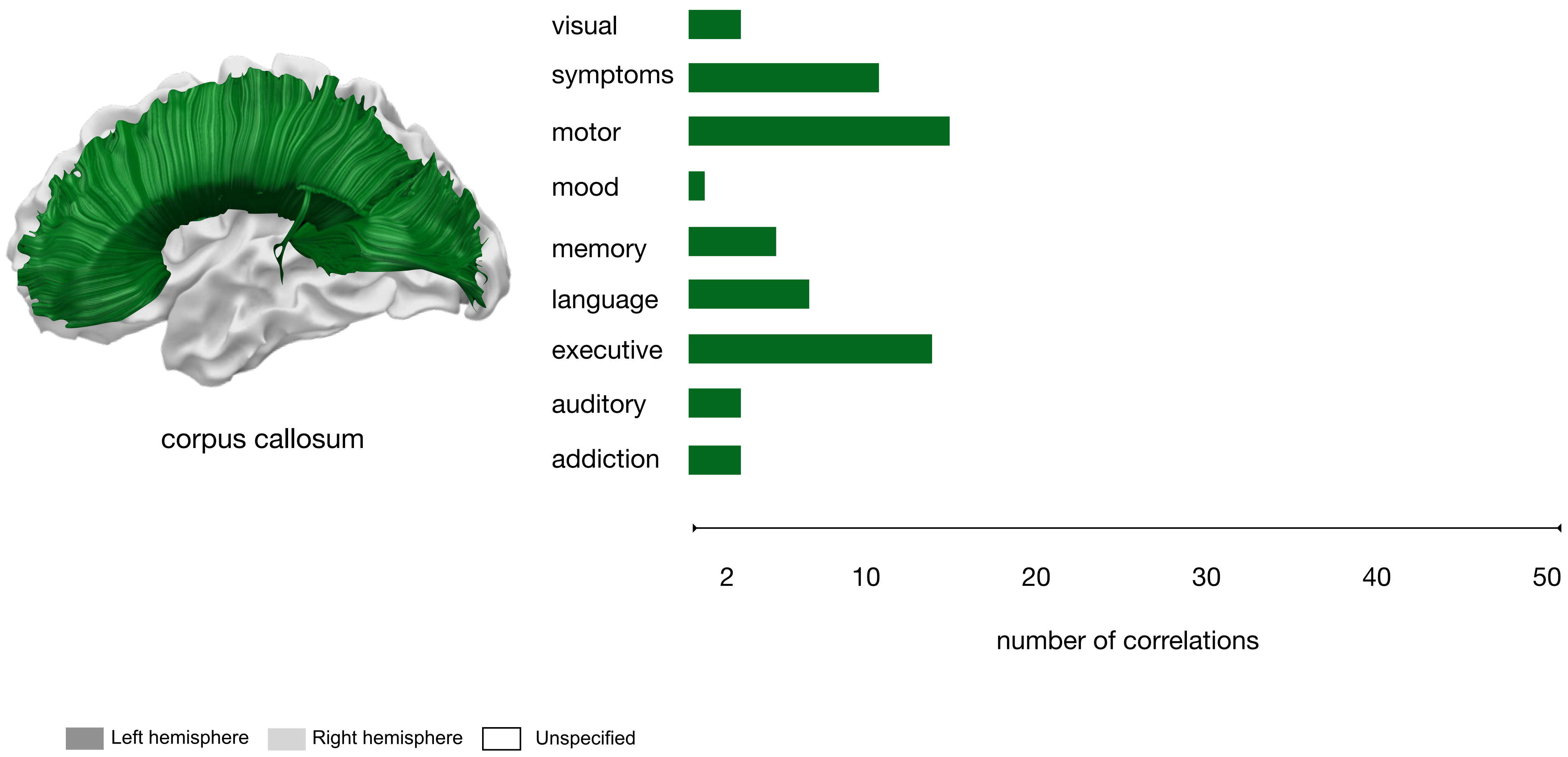
corpus callosum
visual
symptoms
motor
mood
memory
language
executive
auditory
addiction
2
10
20
30
40
50
number of correlations
Left hemisphere
Right hemisphere
Unspecified

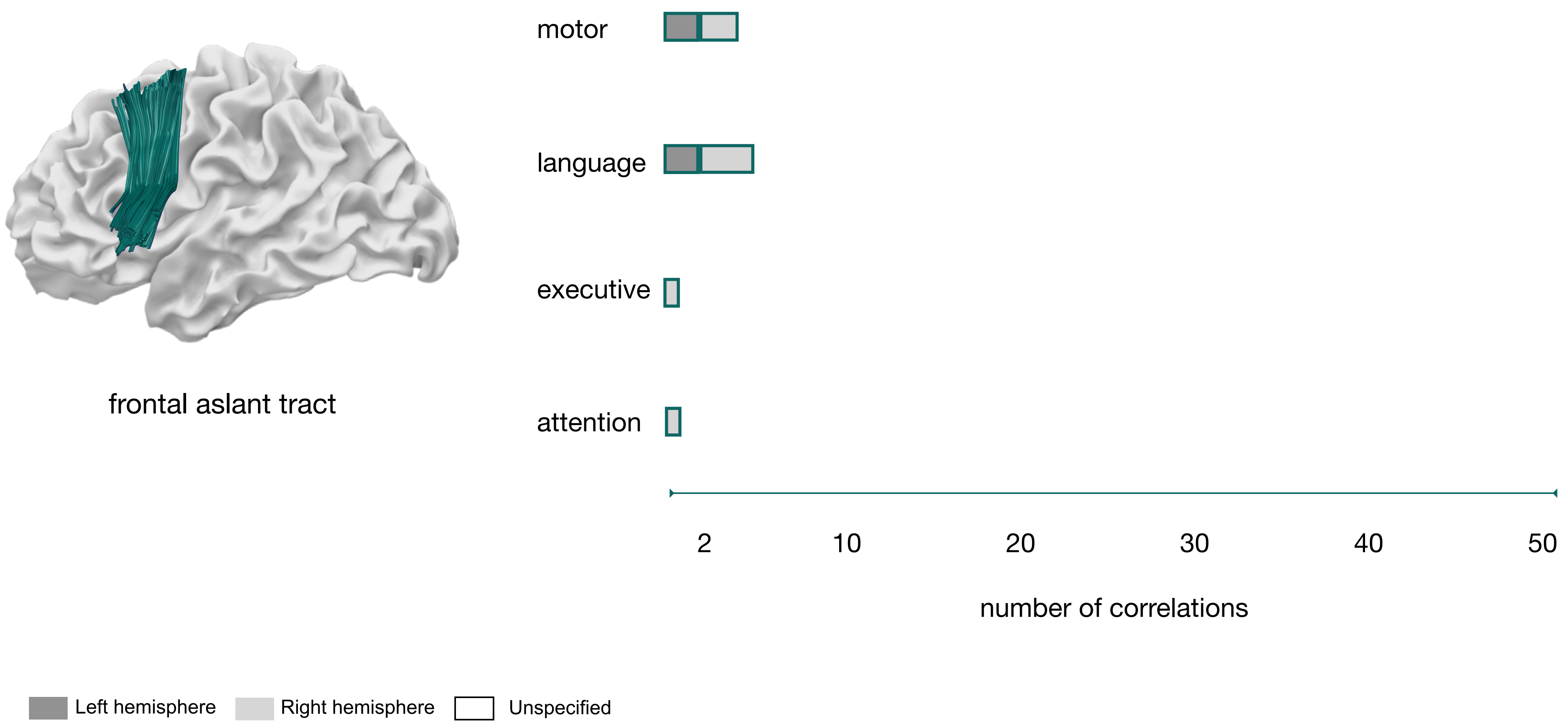

frontal aslant tract
motor
language
executive
attention
2
10
20
30
40
50
number of correlations
Left hemisphere
Right hemisphere
Unspecified

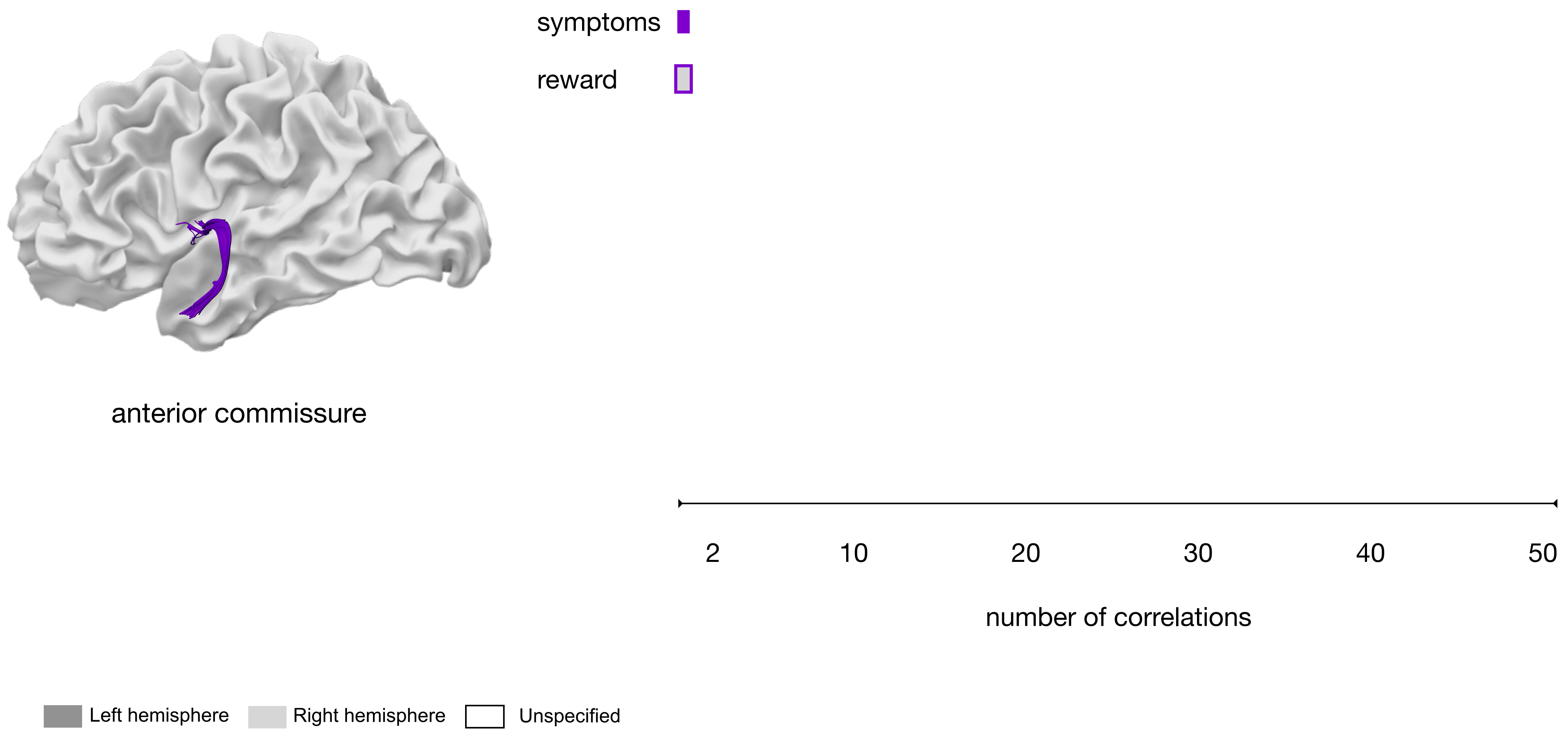
symptoms
reward
anterior commissure
2
10
20
30
40
50
number of correlations
Left hemisphere
Right hemisphere
Unspecified

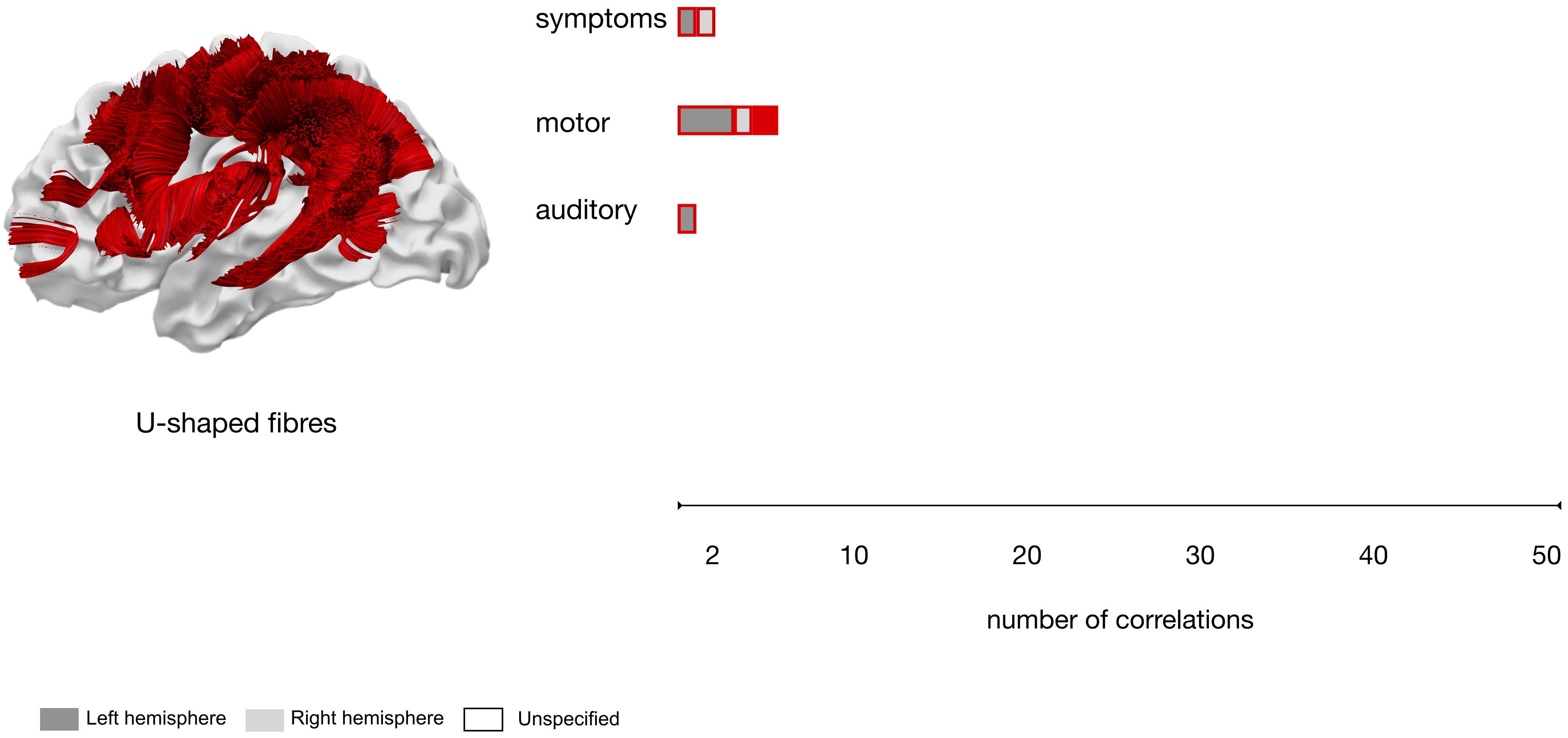
symptoms
motor
auditory
U-shaped fibres
2
10
20
30
40
50
number of correlations
Left hemisphere
Right hemisphere
Unspecified

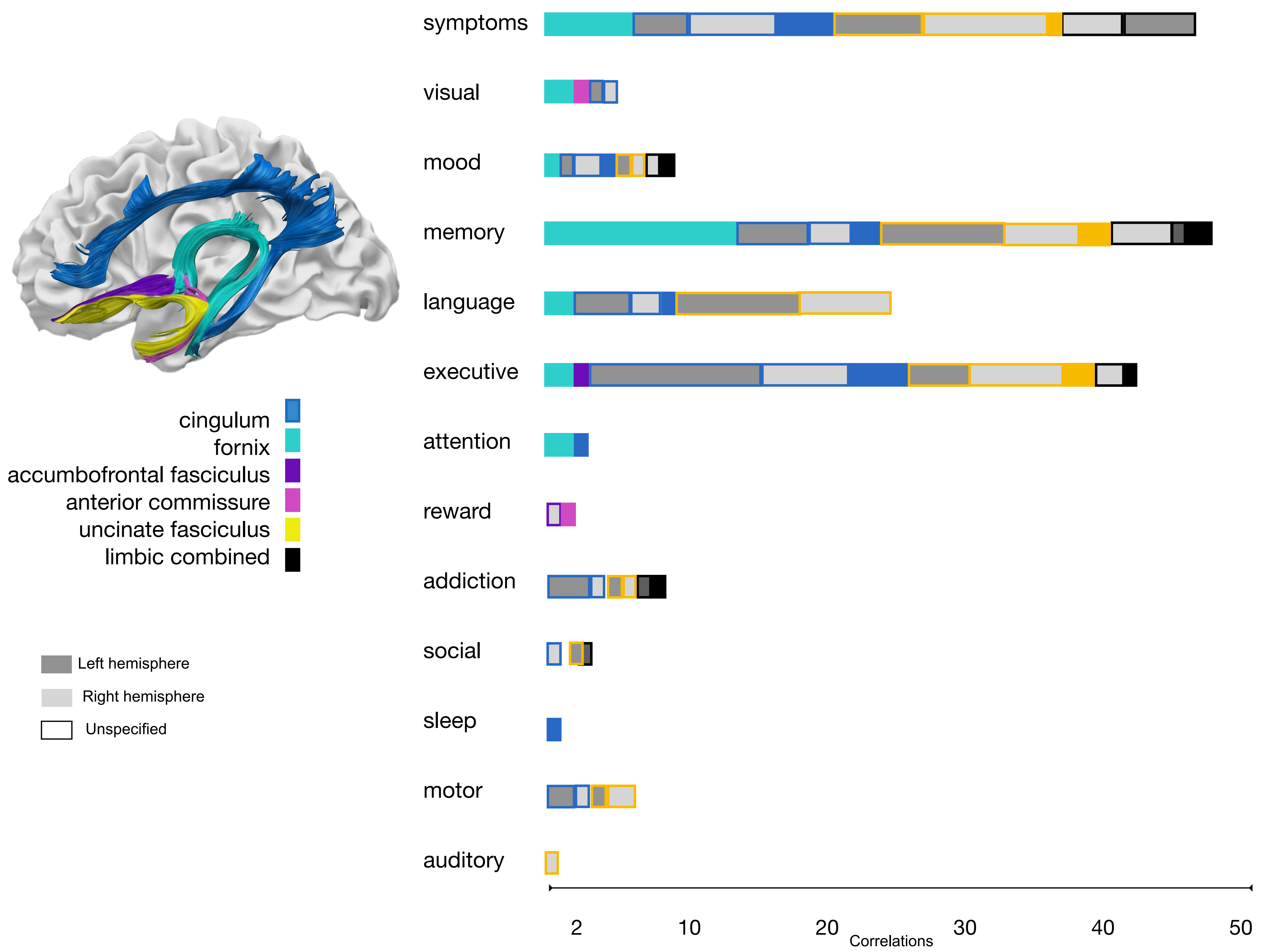
symptoms
visual
mood
memory
language
executive
attention
reward
addiction
social
sleep
motor
auditory
cingulum
fornix
accumbofrontal fasciculus
anterior commissure
uncinate fasciculus
limbic combined
Left hemisphere
Right hemisphere
Unspecified
2
10
20
30
40
50
Correlations

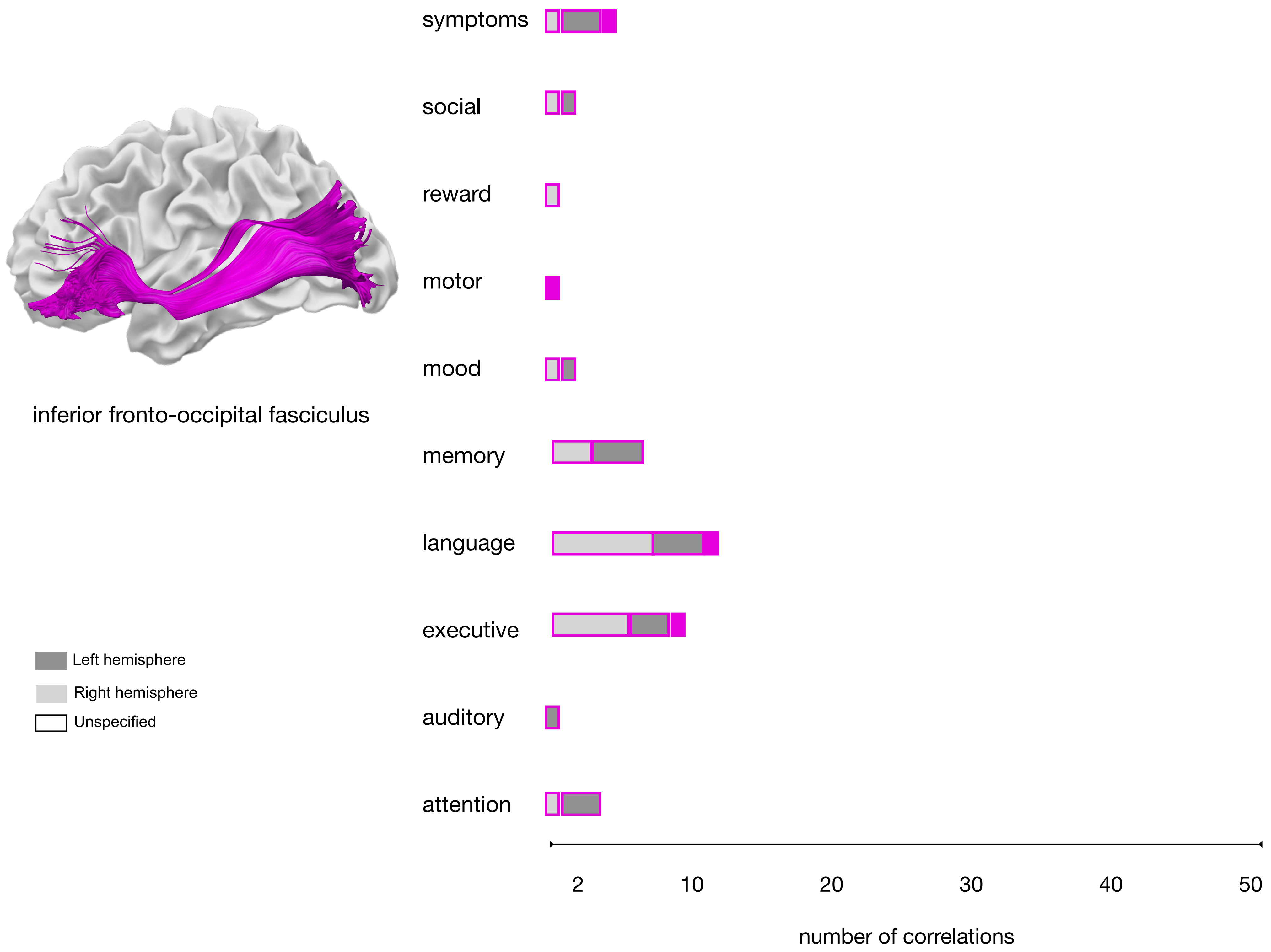

symptoms
social
reward
motor
mood
memory
language
executive
auditory
attention
inferior fronto-occipital fasciculus
Left hemisphere
Right hemisphere
Unspecified
2
10
20
30
40
50
number of correlations

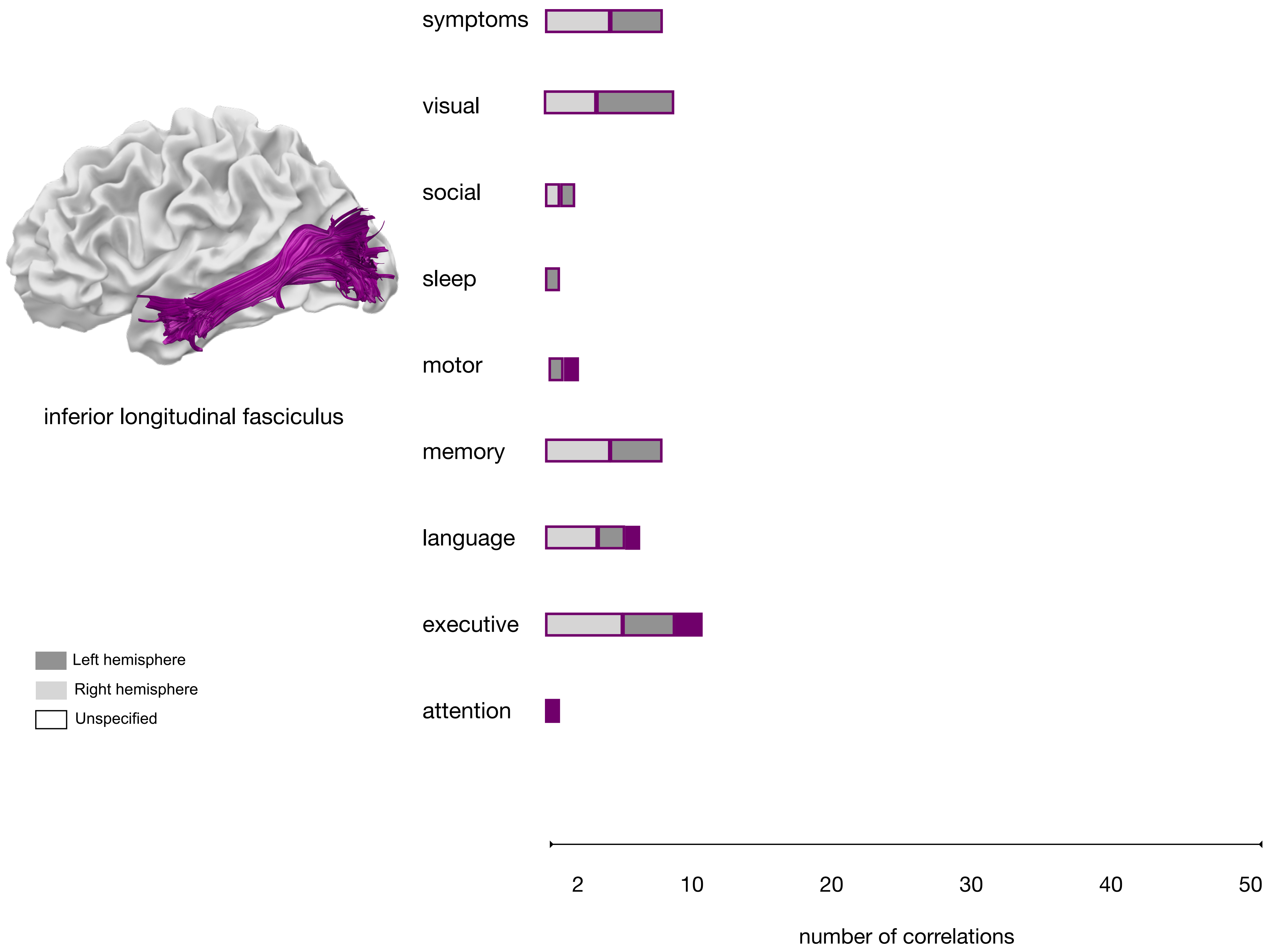
inferior longitudinal fasciculus
symptoms
visual
social
sleep
motor
memory
language
executive
attention
Left hemisphere
Right hemisphere
Unspecified
2
10
20
30
40
50
number of correlations

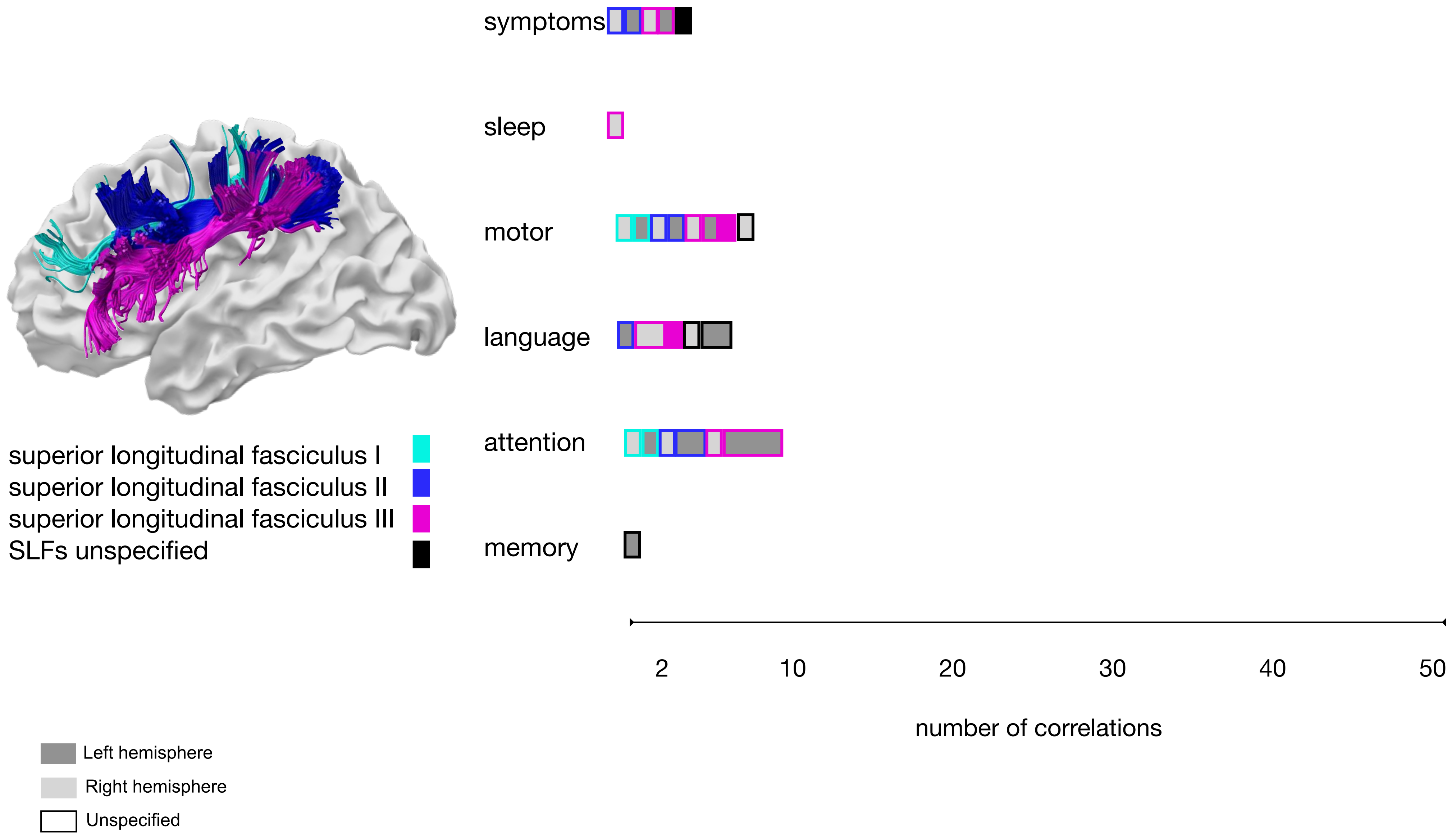

symptoms
sleep
motor
language
attention
memory
superior longitudinal fasciculus I
superior longitudinal fasciculus II
superior longitudinal fasciculus III
SLFs unspecified
2
10
20
30
40
50
number of correlations
Left hemisphere
Right hemisphere
Unspecified

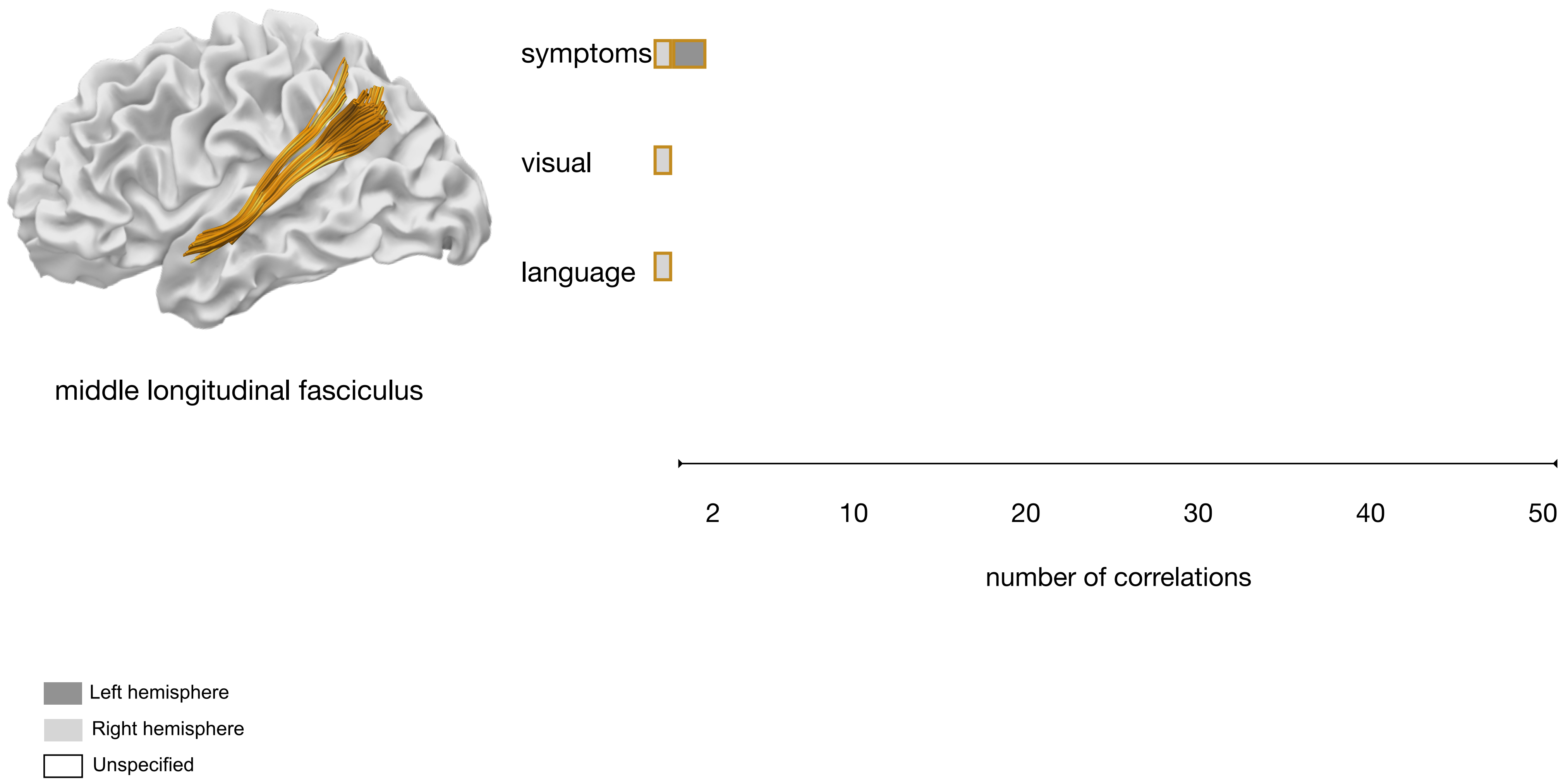
symptoms
visual
language
middle longitudinal fasciculus
2
10
20
30
40
50
number of correlations
Left hemisphere
Right hemisphere
Unspecified

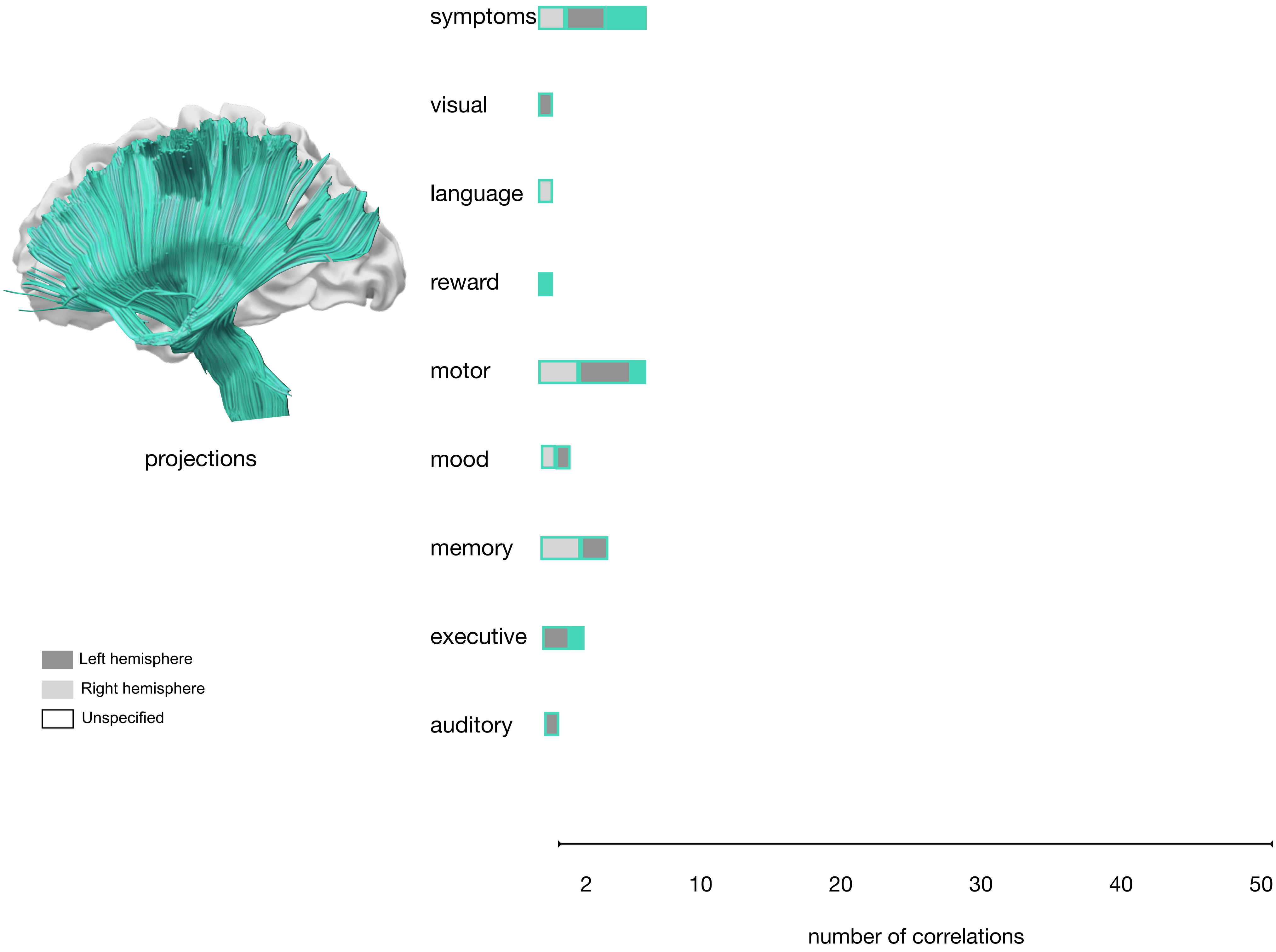

symptoms
visual
language
reward
motor
mood
memory
executive
auditory
projections
Left hemisphere
Right hemisphere
Unspecified
2
10
20
30
40
50
number of correlations

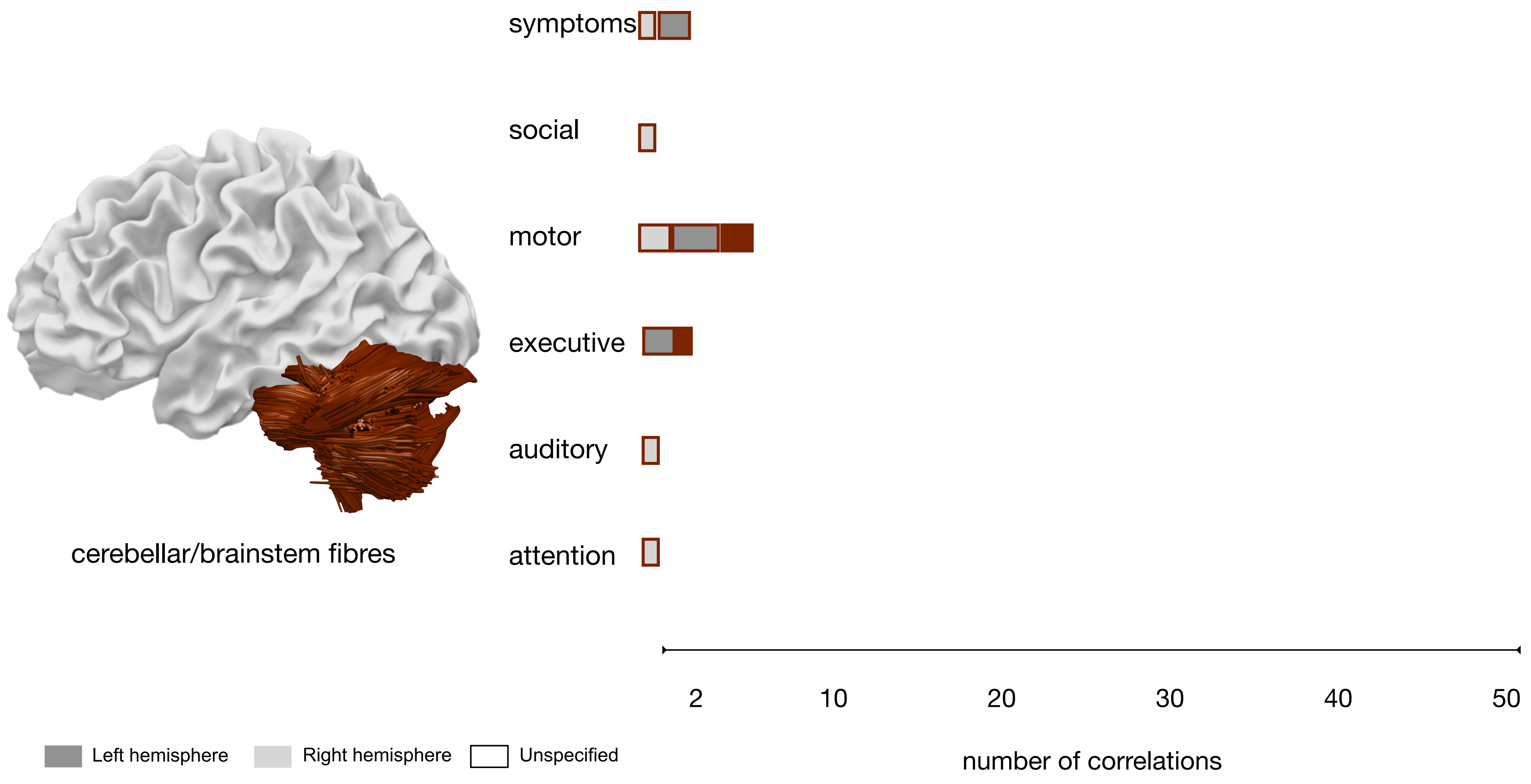
symptoms
social
motor
executive
auditory
attention
cerebellar/brainstem fibres
2
10
20
30
40
50
number of correlations
Left hemisphere
Right hemisphere
Unspecified

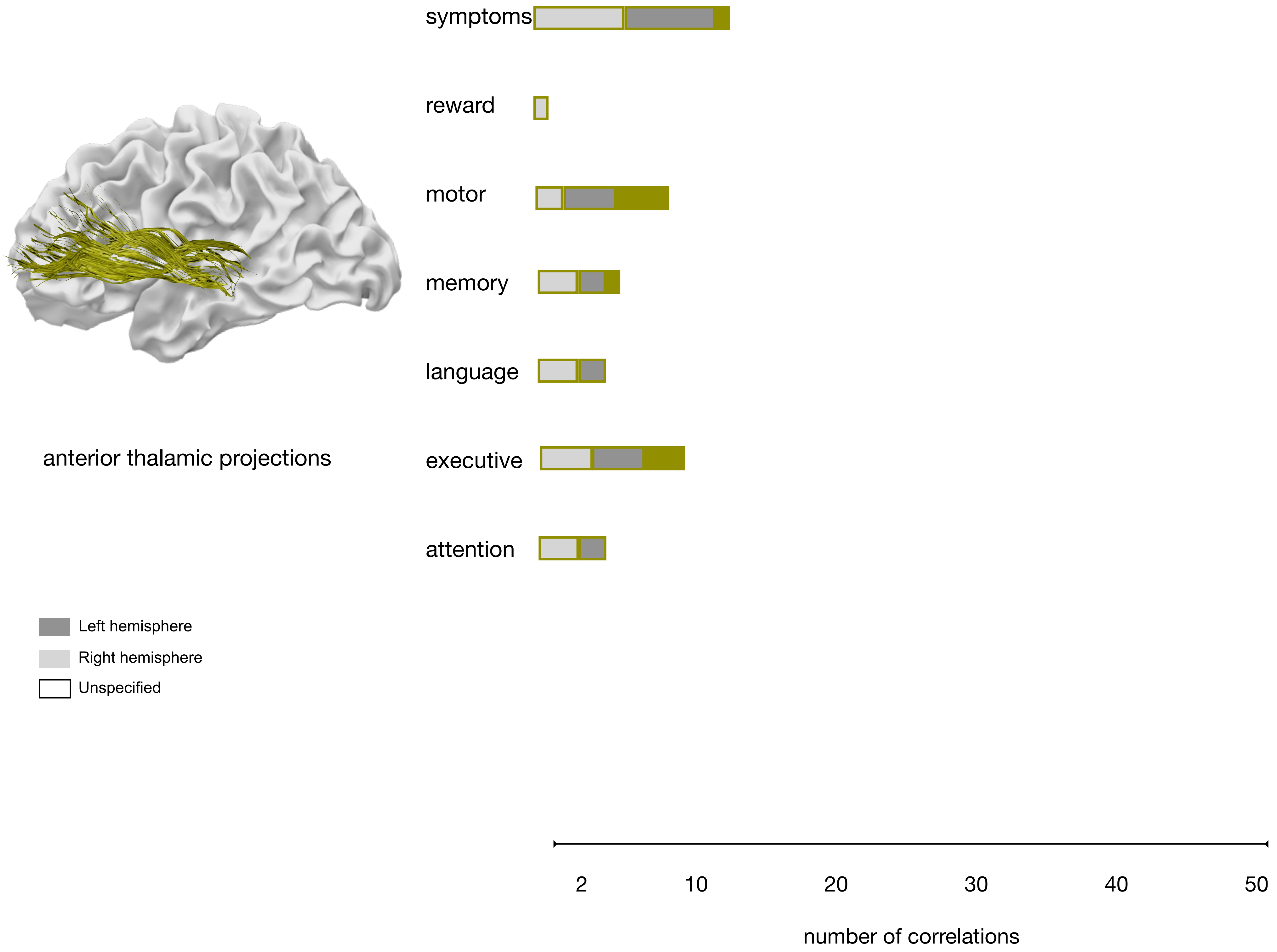
symptoms
reward
motor
memory
language
executive
attention
anterior thalamic projections
Left hemisphere
Right hemisphere
Unspecified
2
10
20
30
40
50
number of correlations

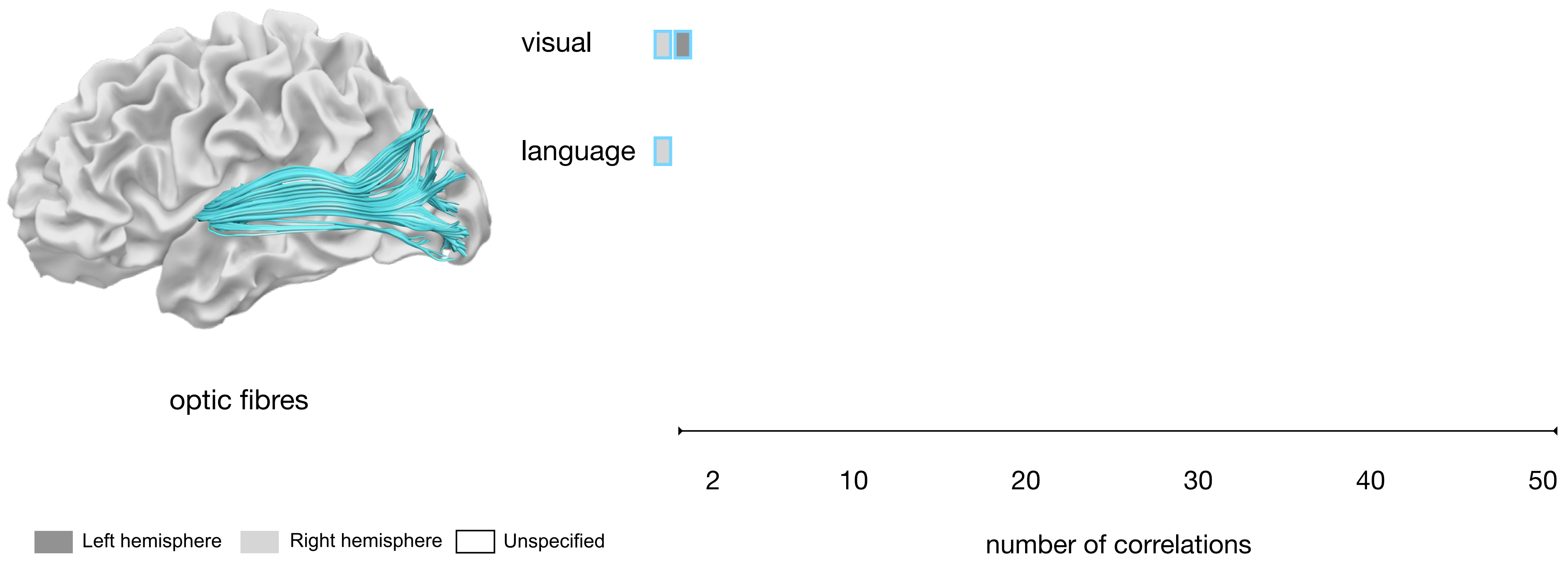
visual
language
optic fibres
2
10
20
30
40
50
number of correlations
Left hemisphere
Right hemisphere
Unspecified

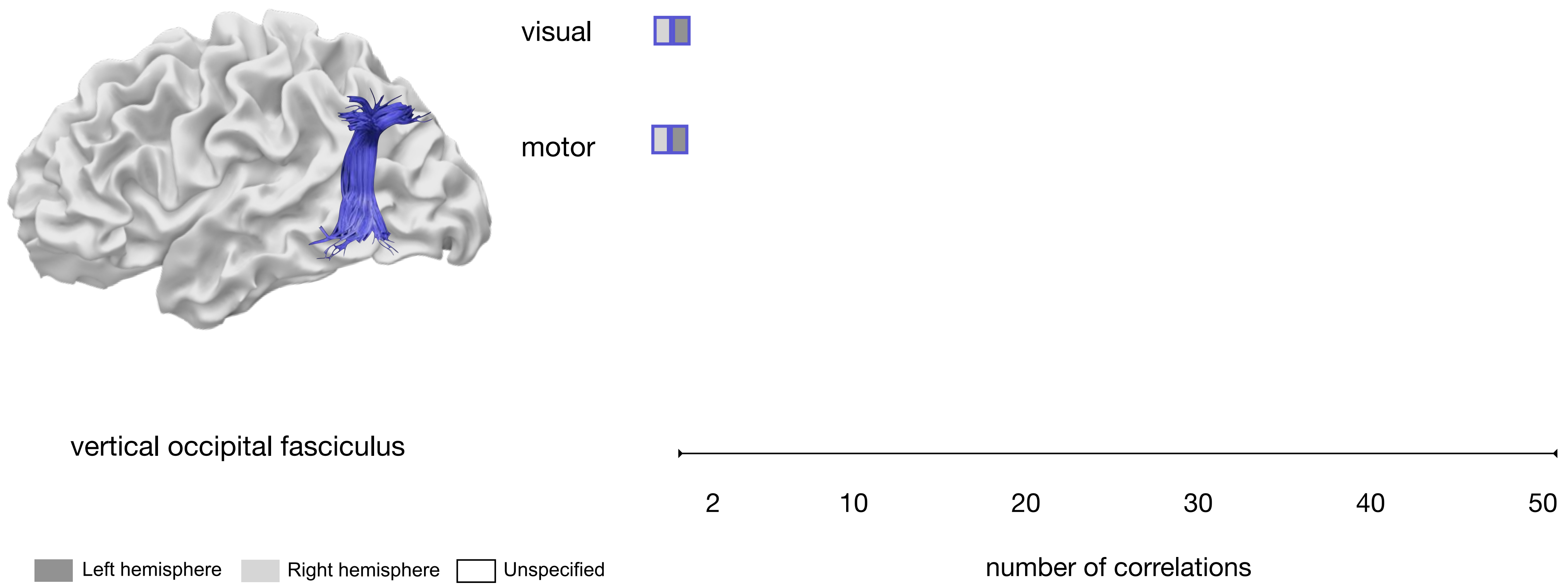
visual
motor
vertical occipital fasciculus
2
10
20
30
40
50
number of correlations
Left hemisphere
Right hemisphere
Unspecified
